# [^177^Lu]Lu-PSMA radioligand therapy in younger prostate cancer patients: A matched-pair analysis between patients ≤ 65 and ≥ 70 years old

**DOI:** 10.1007/s00259-026-07817-2

**Published:** 2026-03-02

**Authors:** Johannes Hornfeck, Paul J. Dahlmann, Sophie C. Siegmund, Franz J. Gildehaus, Astrid Delker, Nina-Sophie Schmidt-Hegemann, Ralph A. Bundschuh, Mathias J. Zacherl, Konrad Klimek, Jozefina Casuscelli, Christian G. Stief, Rudolf A. Werner, Liam Widjaja

**Affiliations:** 1 Department of Nuclear Medicine, LMU University Hospital, LMU Munich, Munich, Germany; 2 Bavarian Cancer Research Center (BZKF), partner site Munich, Munich, Germany; 3 Department of Radiation Oncology, LMU University Hospital, LMU Munich, Munich, Germany; 4 Department of Nuclear Medicine, University Hospital Carl Gustav Carus Dresden, Dresden, Germany; 5 Institute of Radiopharmaceutical Cancer Research, Helmholtz Zentrum Dresden-Rossendorf (HZDR), Rossendorf, Germany; 6 German Cancer Consortium (DKTK), Partner Site Dresden, Dresden, Germany; 7 National Center for Tumor Diseases (NCT), NCT/UCC Dresden, a partnership between DKFZ, Faculty of Medicine and University Hospital Carl Gustav Carus, TUD Dresden University of Technology, and Helmholtz-Zentrum Dresden-Rossendorf (HZDR), Dresden, Germany; 8 Department of Urology, LMU University Hospital, LMU Munich, Munich, Germany; 9 German Cancer Consortium (DKTK), partner site Munich, a partnership between DKFZ and LMU University Hospital Munich, Munich, Germany

**Keywords:** Prostate carcinoma, PSMA, Radioligand therapy, Age-related outcome

## Abstract

**Purpose:**

In prostate cancer, Lutetium-177 ([^177^Lu]Lu )-Prostate-specific membrane antigen (PSMA) radioligand therapy (RLT) shows benefit even in earlier stages of the treatment course, potentially leading to broader application also in younger patients. However, evidence regarding its efficacy and safety in this population remains limited.

**Methods:**

Based on Gleason score, baseline prostate specific antigen (PSA) and prior chemotherapy status, 51 patients ≤ 65 years of age were matched to 51 patients ≥ 70 years. All patients were scheduled for [^177^Lu]Lu-PSMA RLT. Efficacy was evaluated using relative PSA changes after two cycles, along with progression-free survival (PFS, defined as time from 1st RLT to ≥ 25% PSA increase from nadir) and overall survival (OS). Safety was assessed by changes in estimated glomerular filtration rate (eGFR), hemoglobin (Hb), white blood cells (WBC) and platelets from baseline to nadir, alongside Common Terminology Criteria of Adverse Events (CTCAE 5.0) grading for chronic kidney disease (CKD), anemia, leukocytopenia and thrombocytopenia.

**Results:**

RLT showed similar efficacy in both age groups with comparable PSA changes after two cycles (younger patients: -8%; Interquartile range [IQR], -55 to 46% versus older subjects: -20%, IQR, -71 to 46%; *P* = 0.766). No significant differences were observed for PFS (*P* = 0.882) and OS (*P* = 0.17). RLT was safe in both age groups, although eGFR, Hb, WBC and platelets declined significantly in both cohorts (*P* ≤ 0.001 each). Younger patients showed slightly better tolerance, with smaller decreases in Hb (*P* = 0.021) and platelets (*P* = 0.013), along with fewer grade 3 events (young vs. old: anemia, 2 vs. 7 and thrombocytopenia, 0 vs. 2).

**Conclusion:**

[^177^Lu]Lu-PSMA RLT is a safe and effective treatment option in patients ≤ 65 years old, demonstrating comparable efficacy and a slightly more favorable safety profile relative to older patients. These findings support the use of [^177^Lu]Lu-PSMA RLT regardless of age, which may be of importance given recent expansion of the indication spectrum.

**Supplementary Information:**

The online version contains supplementary material available at 10.1007/s00259-026-07817-2.

## Introduction

Lutetium-177 prostate-specific membrane antigen radioligand therapy ([^177^Lu]Lu-PSMA RLT) is an established, safe, and effective option for patients with metastatic castration-resistant prostate cancer (mCRPC) [1]. Its use was initially limited to individuals with end-stage mCRPC [1]. For example, the randomized phase 3 VISION study, which led to the approval of [^177^Lu]Lu-PSMA RLT, enrolled patients who had progressed after at least one androgen receptor pathway inhibitor (ARPI) and taxane-based chemotherapy, resulting in a predominantly older, heavily pretreated population [1]. Consequently, clinical evidence of safety and efficacy in an older population has been widely demonstrated [2, 3]. For instance, *Tauber* et al. reported on their experience of 80 patients over the age of 80 under RLT [2] and demonstrated high efficacy with a PSA decline ≥ 50% achieved in 46% of patients, along with an acceptable safety profile of only 14% grade 3 toxicities [2].

More recently, growing evidence has supported the earlier integration of [^177^Lu]Lu-PSMA RLT in the treatment course [4–6]. *Hofman* et al. compared the efficacy and safety of [^177^Lu]Lu-PSMA RLT with cabazitaxel as second-line chemotherapy [4]. Notably, RLT was associated with a higher PSA response rate (65% vs. 37% with cabazitaxel) and a lower incidence of grade 3–4 toxicities (33% vs. 53%, respectively) [4]. In addition, the PSMAfore trial confirmed clinical benefit of RLT in a chemotherapy-naïve setting, demonstrating superior efficacy and a more favorable toxicity profile compared with an ARPI-switch [5]. Subsequently, the United States Food and Drug Administration recently endorsed the use of [^177^Lu]Lu-PSMA RLT before chemotherapy [7]. Of note, initial results from the PSMAddition trial suggest that [^177^Lu]Lu-PSMA RLT may also provide clinical benefit in the hormone-sensitive disease setting, thereby extending its potential utility beyond castration-resistant prostate cancer [6]. These findings collectively argue for broader, earlier application of RLT, including younger patients.

However, evidence regarding younger individuals remains limited. For example, although the largest phase 3 study, VISION, enrolled patients down to 52 years of age, the study population was predominantly older, with a median age of 71 [1]. Notably, some reports even suggest reduced efficacy in this group [8, 9]. *Hartrampf* et al., for instance, found out that higher age correlated positively with the PSA response in both uni- and multivariate analysis [9], raising concerns about treatment effectiveness in younger patients.

In this study, we therefore sought to evaluate the clinical utility of [^177^Lu]Lu-PSMA RLT in patients ≤ 65 years old when compared to individually matched patients ≥ 70 years old, focusing on efficacy and safety.

## Materials and methods

### Patient population and matching criteria

This retrospective, single-center study screened a large cohort of 298 patients who were treated with [^177^Lu]Lu-PSMA radioligand therapy between September 2014 and January 2025 as part of routine clinical care after disease progression following multiple prior therapies, including enzalutamide, abiraterone, and chemotherapy. From this cohort, we identified 51 patients aged ≤ 65 years. For each of these younger patients, a matched comparator aged ≥ 70 years was individually selected from the remaining cohort. Matching criteria were based on clinically relevant prognostic factors [10] and were adapted from a previous study [11]. In this regard, for each patient ≤ 65 years old, a corresponding patient ≥ 70 years old was identified with similar Gleason score (classes: 6–7, 8–10, undefined), baseline prostate specific antigen (PSA, classes: 0–50 ng/ml, 50–200 ng/ml, 200–600 ng/ml and > 600 ng/ml) and prior chemotherapy (Table 1).

**Table 1:** Patient characteristics

Variable (mean ± SD)	≤ 65 (*n* = 51)	≥ 70 (*n* = 51)	*P*
Age	59 ± 4	77 ± 5	< ***0.001***
Gleason score	8 ± 1	9 ± 1	0.541
Previous treatments (%)			
Radical prostatectomy	55	57	0.844
Primary radiation therapy	4	8	0.405
Androgen deprivation therapy	94	100	0.083
Enzalutamide	51	69	0.070
Abiraterone acetate	53	80	***0.003***
Previous chemotherapy	75	75	1
Docetaxel	73	73	1
Cabazitaxel	16	18	0.793
Interval from initial diagnosis to RLT (d)	2622 ± 6134	3041 ± 2128	0.647
Standard laboratory value (mean ± SD)			
Hb (g/dl)	12.1 ± 1.9	11.8 ± 1.6	0.265
WBC (×10^3^/µL)	6.4 ± 2.1	6.6 ± 2	0.495
Platelets (×10^3^/µL)	263 ± 78	271 ± 102	0.688
eGFR (ml/min/1.73m^2^)	94 ± 15	76 ± 15	***< 0.001***
AST (U/I)	34 ± 15	31 ± 17	0.353
ALT (U/I)	22 ± 11	17 ± 11	***0.009***
Bilirubin (mg/dL)	0.44 ± 0.31	0.86 ± 3.03	0.325
AP (U/I)	171 ± 151	191 ± 295	0.573
LDH (U/I)	331 ± 165	305 ± 131	0.402
PSA (µg/L)	196 ± 297	196 ± 310	0.994
Site of tumor lesions (%)			
Osseous	90	86	0.543
Lymph nodes	76	73	0.653
Hepatic	12	2	0.051
Prostate bed	43	37	0.549

Significant parameters are marked in bold and italic. *SD* standard deviation, *RLT* radioligand therapy, *Hb* hemoglobin, *WBC* white blood cells, *eGFR* estimated glomerular filtration rate, *AST* aspartate transaminase, *ALT* alanine transaminase, *AP* alkaline phosphatase, *LDH* lactate dehydrogenase, *PSA* prostate-specific antigen

The institutional review board (25–0130) reviewed and approved this study, waiving the requirement for informed consent to scientific analysis. Parts of this cohort have been previously published, though without an age-focused analysis [12–17].

### [^177^Lu]Lu-PSMA RLT and clinical outcome

Patients received an average of 3.5 ± 2 RLT cycles (3.6 ± 2.3 cycles in patients ≤ 65 years old versus 3.5 ± 1.7 cycles in patients ≥ 70 years old, *P* = 0.736). RLT was performed following the German Medicinal Products Act, AMG § 13.2b, the Declaration of Helsinki and in accordance to the joint procedure guidelines of the European Association of Nuclear Medicine and the Society of Nuclear Medicine and Molecular Imaging [18]. In all patients, PSMA-avid disease was confirmed using PSMA-targeted imaging, e.g., Fluor-18 ([^18^F]F)-PSMA-1007 or Gallium-68 ([^68^Ga]Ga)-PSMA-11 positron emission tomography/computed tomography (PET/CT). RLT was carried out using either [^177^Lu]Lu-PSMA-617 (51% of all cycles) or [^177^Lu]Lu-PSMA-I&T (49% of all cycles). Per cycle, 7.2 ± 1.1 GBq were given intravenously.

### Assessment of clinical outcome

At and between each cycle, laboratory values including PSA for response evaluation along with estimated glomerular filtration rate (eGFR), hemoglobin (Hb), white blood cells (WBC) and platelet count were assessed. Treatment response after two cycles was assessed according to Prostate Cancer Clinical Trial Working Group (PCWG 3) [19]. Thus, a PSA increase ≥ 25% was classified as progressive disease (PD), a PSA changes between − 50 and 25% as stable disease (SD) and a PSA decrease ≥ 50% as partial response [19]. In patients ceasing RLT before the follow-up after the 2nd cycle, last available PSA-value was applied for response evaluation. Progression-free survival (PFS) was defined as the time from first RLT to a ≥ 25% rise in PSA from nadir. Overall survival (OS) was defined as the time between first RLT and death. Safety was assessed following Common Terminology Criteria of Adverse Events (CTCAE 5.0) for chronic kidney disease (CKD), anemia, leukocytopenia und thrombocytopenia [20].

### Statistical analysis

Statistical analysis was carried out using GraphPadPrism 10 (GraphPad Software, San Diego, CA, USA) and SPSS Statistics 31 Inc. (IBM; Chicago, IL, USA). Unpaired t-test and Fisher’s exact test were applied to test whether there were any differences in baseline characteristics, treatment response after two cycles or relative changes of laboratory values between both age groups. In addition, paired t-test was used to evaluate changes in laboratory values from baseline to nadir. Finally, we performed Kaplan-Meier analysis and log-rank test to compare PFS and OS in both age groups. A *P* value smaller than 0.05 was considered as statistically significant.

## Results

### Baseline characteristics of both age groups

After matching for PSA, Gleason Score and prior chemotherapy, baseline patient characteristics of patients ≤ 65 years old (age, 59 ± 4) and patients ≥ 70 years old (age, 77 ± 5) were largely comparable (Table 1). Prior to RLT, older patients were more likely to have received abiraterone acetate and eGFR was lower in this cohort (*P* ≤ 0.003). Younger patients had higher alanine transaminase (ALT) (*P* = 0.009), while frequency of hepatic metastases did not reach significance (12% versus 2% in patients ≥ 70 years old, *P* = 0.051).

### Comparison of efficacy

Following two cycles, we observed comparable response to [^177^Lu]Lu-PSMA RLT in both age groups (Table 2). Median PSA change was − 8% (Interquartile range [IQR], −55 to 46%) in younger patients and − 20% (IQR, −71 to 46%) in older patients (*P* = 0.766). Among younger individuals, 18 (35%) had PD, 19 (37%) had SD and 14 (27%) had PR. Similar distribution (PD, 16 [31%]; SD, 19 [37%]; PR, 16 [31%]) was observed in patients ≥ 70 years old (*P* ≥ 0.828).

**Table 2:** Response after two cycles RLT

Variable	≤ 65 (*n* = 51)	≥ 70 (*n* = 51)	*P*
PSA change (%, median [IQR])	−8% (−55 to 46%)	−19% (−71 to 46%)	0.766
Progressive Disease (n [%])	18 (35%)	16 (31%)	0.834
Stable Disease (n [%])	19 (37%)	19 (37%)	1
Partial Response (n [%])	14 (27%)	16 (31%)	0.828

*RLT* radioligand therapy, *PSA* prostate-specific antigen, *IQR* Interquartile range

**Fig. 1:**
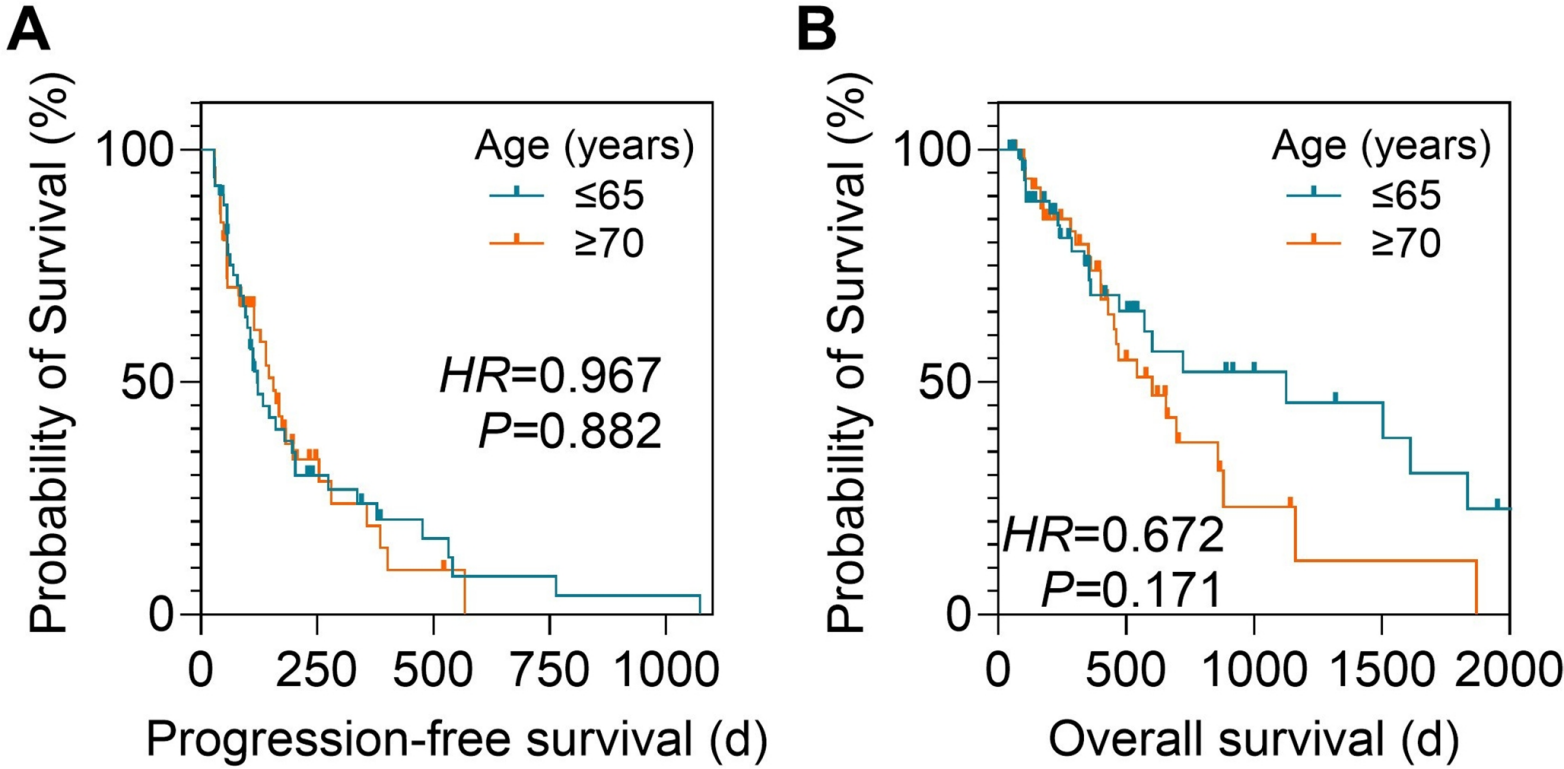
Kaplan-Meier analysis of Progression-free survival (PFS) and Overall survival (OS). RLT demonstrated comparable effectiveness in patients ≤ 65 years and ≥ 70 years old, with no statistically significant differences in PFS (**A**) or OS (**B**)

No significant differences in PFS and OS were identified between both age groups. Median PFS was 121 days in patients ≤ 65 years old and 156 days in patients ≥ 70 years old (hazard ratio [HR], 0.967; *P* = 0.882; Fig. 1A). Median OS was 1125 days in patients ≤ 65 years old and 603 days in patients ≥ 70 years old (Hazard ratio [HR], 0.672; *P* = 0.171; Fig. 1B).

### Comparison of safety

**Fig. 2:**
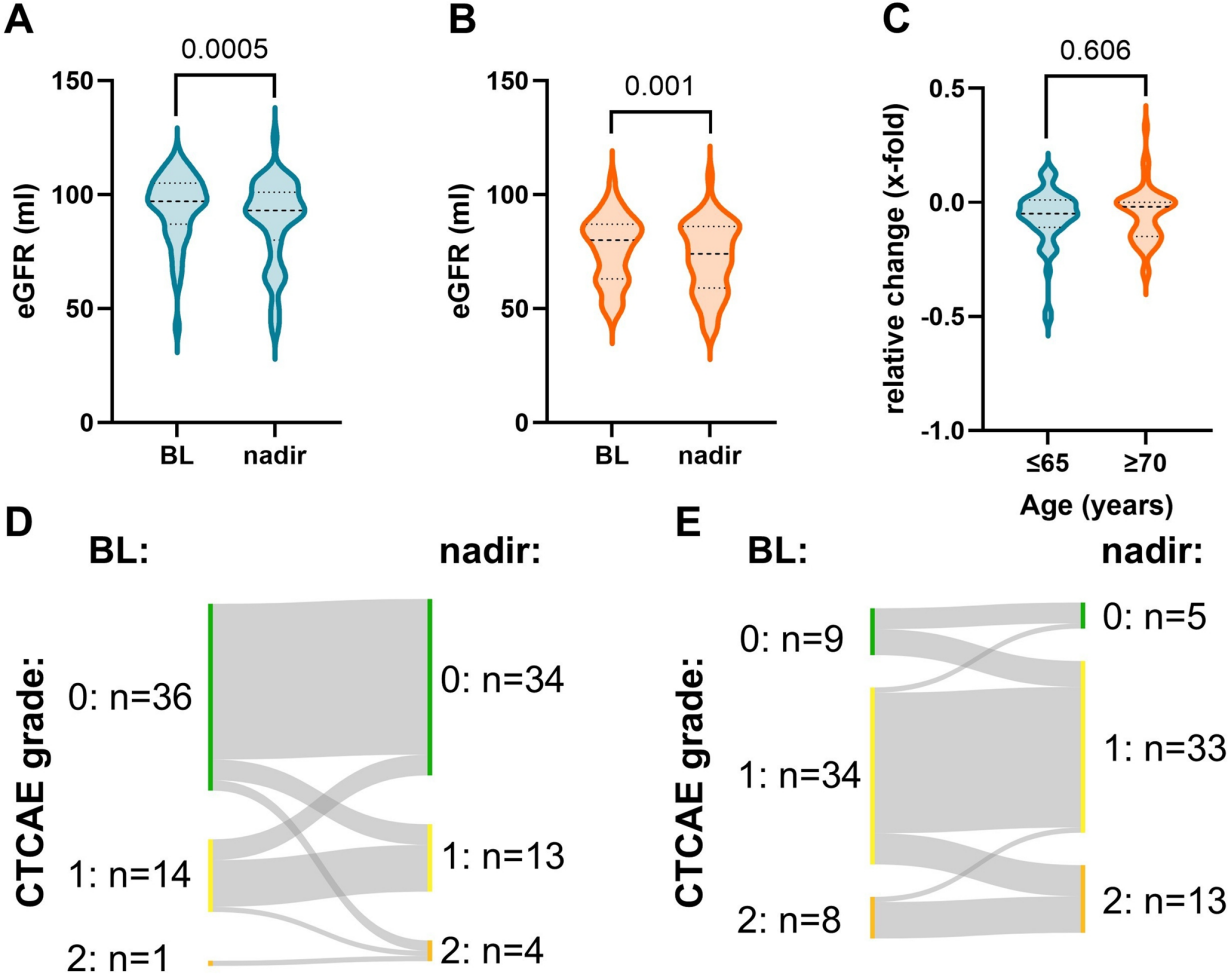
Chronic kidney disease (CKD) in patients ≤ 65 years and ≥ 70 years old under radioligand therapy (RLT). Violin plots showing the baseline estimated glomerular filtration rate (eGFR) and the nadir during RLT for patients ≤ 65 years (**A**) and ≥ 70 years old (**B**), along with a direct comparison of the relative changes (**C**). Sankey diagrams illustrating the distribution of CKD grades, classified according to common terminology criteria of adverse events (CTCAE), before and during RLT for patients ≤ 65 years (**D**) and ≥ 70 years old (**E**). During RLT, eGFR declined in both age groups (**A**,** B**) with similar changes being observed in both patients ≤ 65 years and ≥ 70 years old (**C**). No cases of grade 3 CKD were reported in either age group **(D**,** E)**

**Fig. 3:**
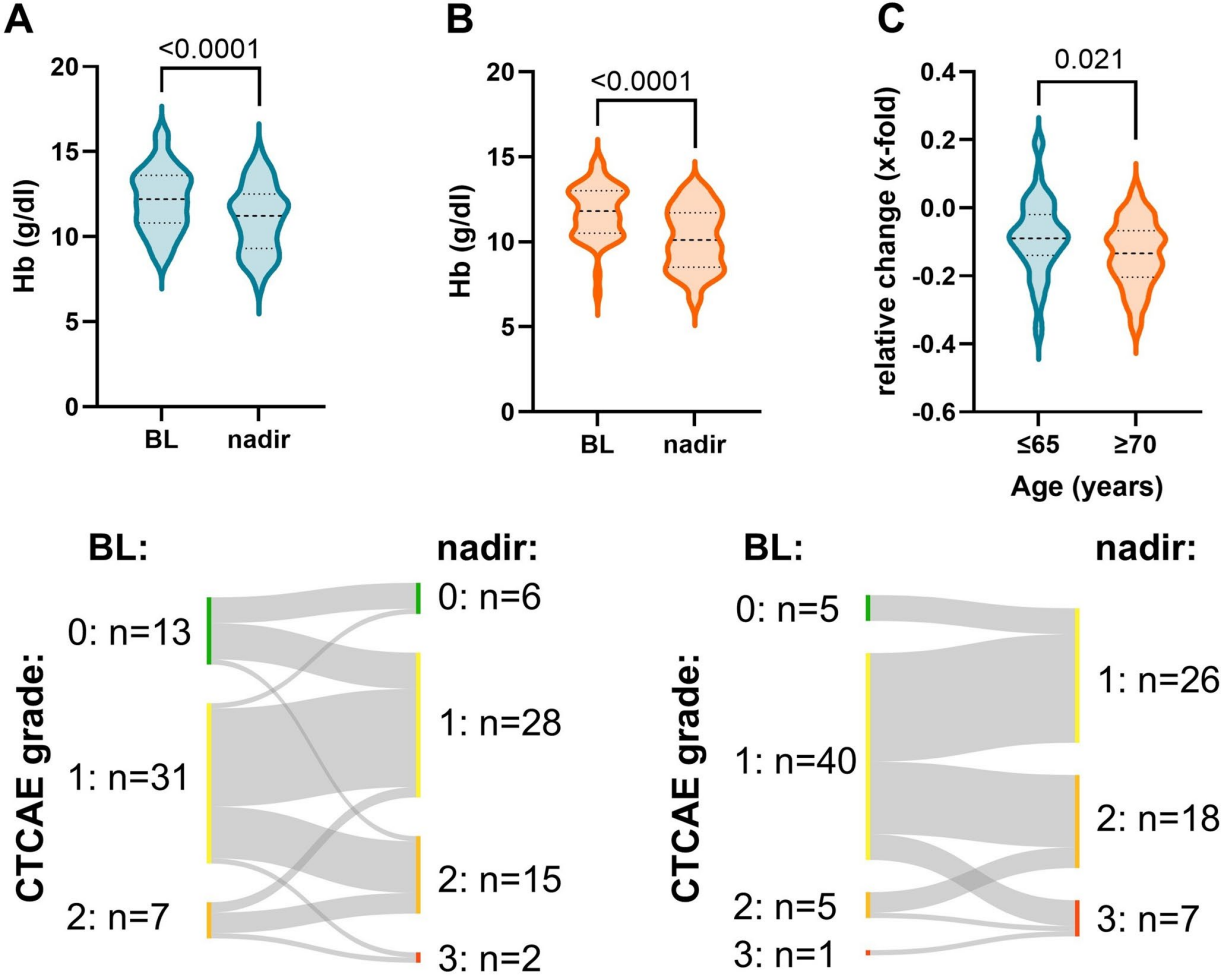
Anemia in patients ≤ 65 years and ≥ 70 years old under radioligand therapy (RLT). Violin plots showing the baseline hemoglobin and the nadir during RLT for patients ≤ 65 years (**A**) and ≥ 70 years old (**B**), along with a direct comparison of the relative changes (**C**). Sankey diagrams illustrating the distribution of anemia grades, classified according to common terminology criteria of adverse events (CTCAE), before and during RLT for patients ≤ 65 years (**D**) and ≥ 70 years old (**E**). During RLT, Hb declined in both age groups (**A**,** B**), however with stronger decreases observed in patients ≥ 70 years old (**C**). In line, grade 3 anemia was more frequent in patients ≥ 70 years old **(D**,** E)**

**Fig. 4:**
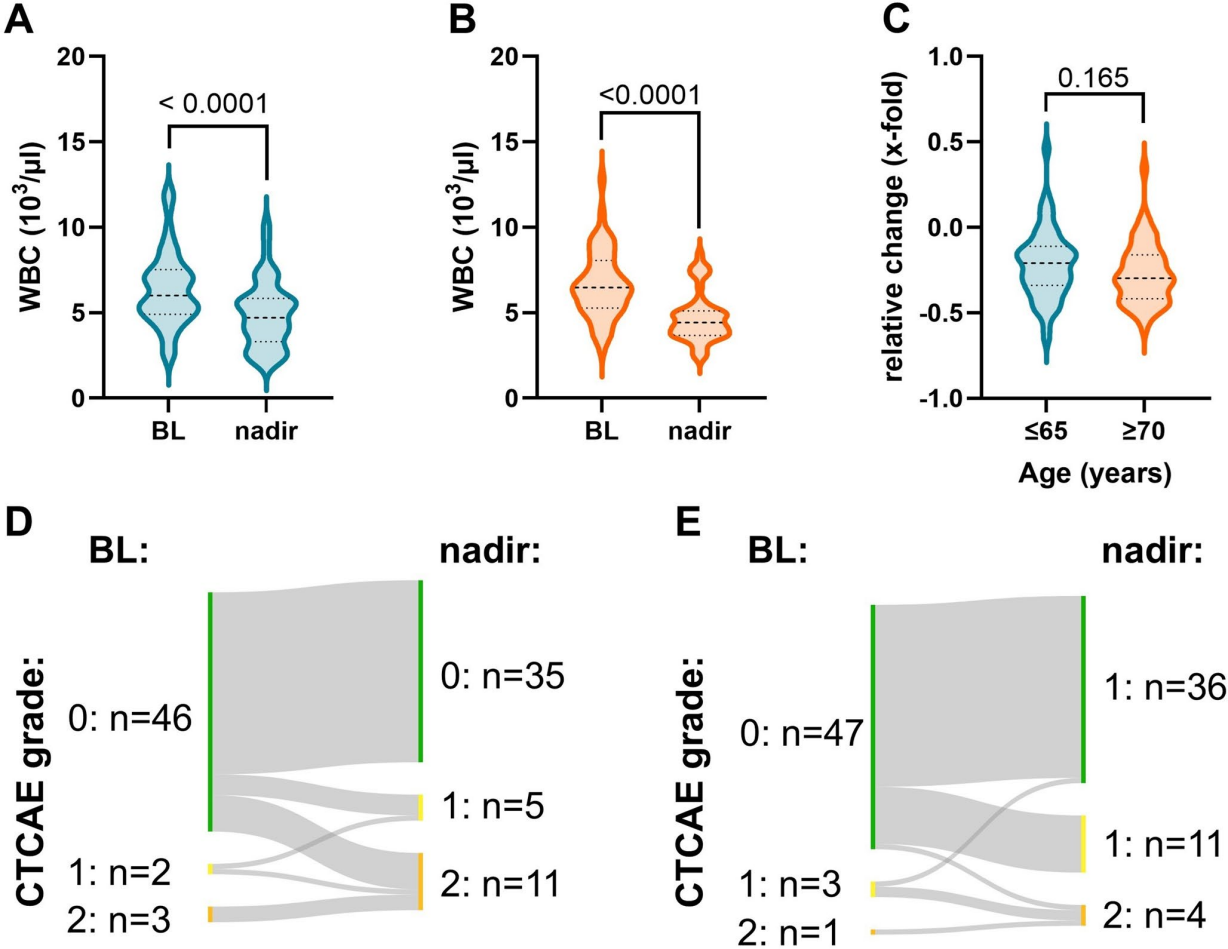
Leukocytopenia in patients ≤ 65 years and ≥ 70 years old under radioligand therapy (RLT). Violin plots showing the baseline white blood cell (WBC) count and the nadir during RLT for patients ≤ 65 years (**A**) and ≥ 70 years old (**B**), along with a direct comparison of the relative changes (**C**). Sankey diagrams illustrating the distribution of leukocytopenia grades, classified according to common terminology criteria of adverse events (CTCAE), before and during RLT for patients ≤ 65 years (**D**) and ≥ 70 years old (**E**). During RLT, WBC declined in both age groups (**A**,** B**) with similar changes being observed in both patients ≤ 65 years and ≥ 70 years old (**C**). No cases of grade 3 leukocytopenia were reported in either age group **(D**,** E)**

**Fig. 5:**
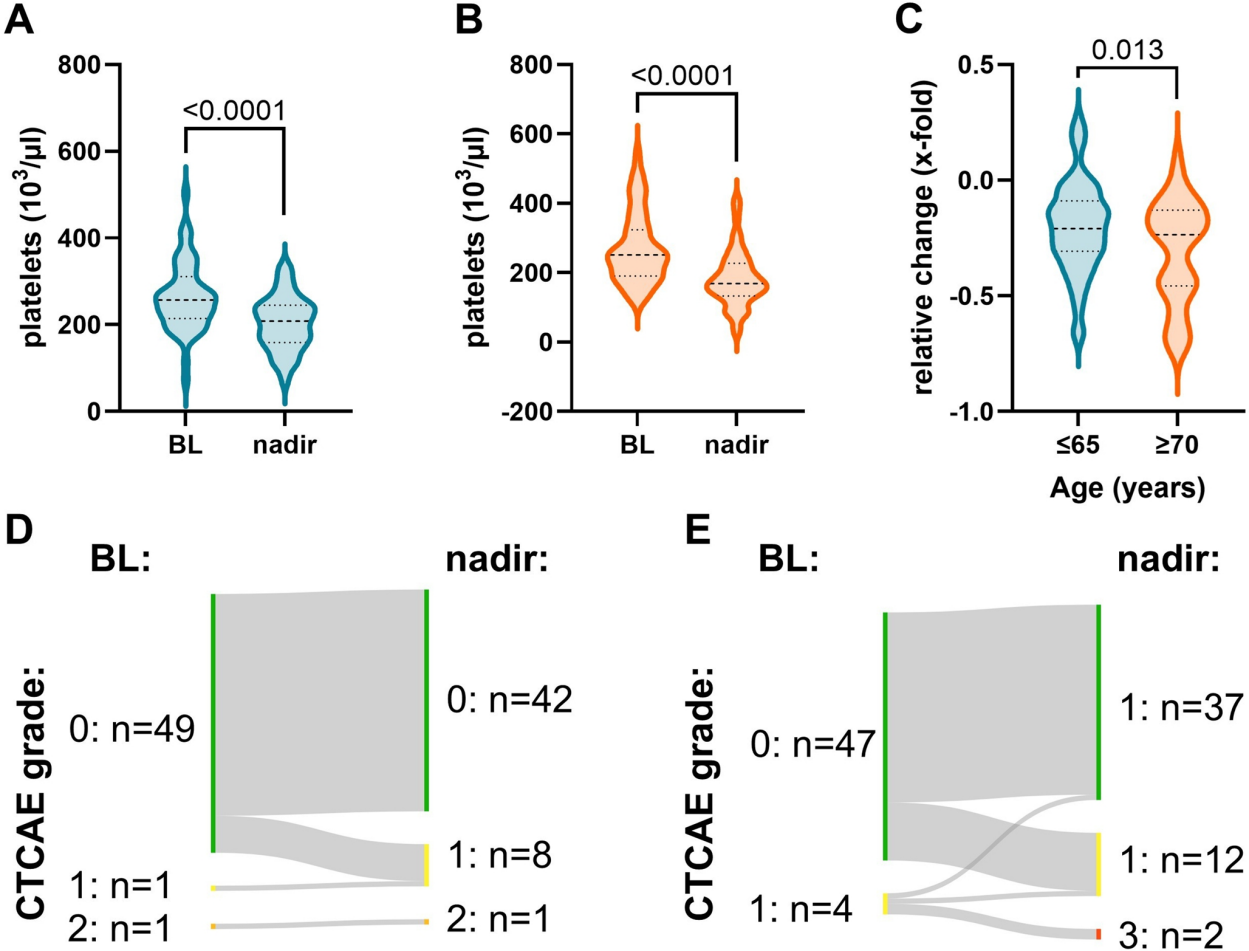
Thrombocytopenia in patients ≤ 65 years and ≥ 70 years old under radioligand therapy (RLT). Violin plots showing the baseline platelet count and the nadir during RLT for patients ≤ 65 years (**A**) and ≥ 70 years old (**B**), along with a direct comparison of the relative changes (**C**). Sankey diagrams illustrating the distribution of thrombocytopenia grades, classified according to common terminology criteria of adverse events (CTCAE), before and during RLT for patients ≤ 65 years (**D**) and ≥ 70 years old (**E**). During RLT, platelets declined in both age groups (**A**,** B**), however with stronger decreases observed in patients ≥ 70 years old (**C**). In line, grade 3 thrombocytopenia was more frequent in patients ≥ 70 years old **(D**,** E)**

[^177^Lu]Lu-PSMA RLT was well tolerated in both groups, with a trend toward a more favorable safety profile in patients aged ≤ 65 years. Significant decreases during RLT were observed for eGFR, Hb, WBC and platelets in both cohorts, albeit with clinically limited relevance (*P* ≤ 0.001; Figs. 2, 3, 4 and 5). There was a tendency toward a more favorable safety profile in patients ≤ 65 years old, with significantly smaller reductions in both Hb (*P* = 0.021; Fig. 3C) and platelets (*P* = 0.013; Fig. 5C). In line, grade 3 toxicities were more common in older patients with 7 cases (14%) of grade 3 anemia (versus 2 [4%] in younger patients; Fig. 3D&E) and 2 cases (4%) of grade 3 thrombocytopenia (versus none in younger patients; Fig. 5D&E). No grade 3 CKD or leukocytopenia, and no grade 4 toxicities of any type occurred in both groups.

## Discussion

To the best of our knowledge, our study compared for the first time the clinical utility of [^177^Lu]Lu-PSMA RLT in younger patients relative to older subjects using a matched-pair design. We demonstrate that patients ≤ 65 years old achieve comparable efficacy to matched patients ≥ 70 years old, while younger individuals exhibiting an even more favorable safety profile. These findings support the use of [^177^Lu]Lu-PSMA RLT regardless of age, which may be of relevance given recent expansion of the indication spectrum.

Evidence supporting an earlier use of PSMA-targeted RLT is accumulating rapidly [4–6]. The TheraP trial established superiority over the second-line chemotherapy cabazitaxel [4], the PSMAfore trial demonstrated utility in chemotherapy-naïve patients [5], and the PSMAddition trial reported on potential benefit even in a hormone-sensitive setting [6]. As RLT moves earlier in the treatment course, younger patients will increasingly receive this therapy. Yet previous reports have suggested that younger age might predict worse outcomes under RLT [8, 9]. However, these studies were not primarily designed to evaluate treatment efficacy in younger patients and lacked sufficient adjustment for key confounding factors, including Gleason score [8–10]. As a result, the reported outcomes may be driven more by underlying tumor aggressiveness than by age itself. Supporting this notion, the time from initial diagnosis to first RLT was also shown to be a significant determinant of outcome in that study [8, 9]. In line, we observed a trend toward higher ALT (*P* = 0.009) and more frequent hepatic metastases in patients ≤ 65 years (12% vs. 2%; *P* = 0.051), a known negative prognostic factor [21]. Nonetheless, performing a dedicated match-paired analysis we found no evidence of reduced treatment efficacy in younger patients, as PSA response after two cycles (*P* = 0.766), PFS (*P* = 0.882), and OS (*P* = 0.171) were comparable. Notably, the time from initial diagnosis to first RLT was also similar in both cohorts, also indicating comparable tumor aggressiveness after matching the cohorts.

Our matching criteria (Gleason score, PSA and previous chemotherapy) were selected based on known prognostic significance and established in a previous study [9, 10]. Thus, both age groups were comparable to a large extend with only minor differences in baseline characteristics, e.g., lower eGFR and higher ALT at baseline in patients ≥ 70 years old. Another relevant prognostic factor under PSMA-targeted RLT is the PSMA-expression in the metastases which can be quantified by whole-body tumor segmentation on PSMA PET/CT [8, 22]. In this regard, *Seifert* et al. demonstrated that high PSMA expression on [^68^Ga]Ga-PSMA PET/CT before treatment initiation is linked to a prolonged overall survival in RLT patients [22]. We were unable to include PET-based biomarkers in our analysis, as imaging modalities, e.g., different [^68^Ga]Ga and [^18^F]F labeled tracers, for baseline staging were inconsistent in our cohort. Nonetheless, future studies should investigate whether PET-based PSMA-expression is comparable between both groups and may also evaluate whether improved patient selection for RLT is independently linked to baseline PET regardless of age.

Regarding safety, [^177^Lu]Lu-PSMA RLT was well tolerated across both age groups. Of note safety profile in patients ≤ 65 years old was even favorable when compared to patients ≥ 70 years old with smaller declines in Hb and platelets, translating in fewer cases of anemia or further underscore the favorable safety profile in younger individuals [2, 3]. For instance, *Schweigert* et al. reported on grade 3 anemia in 6% (versus 4% in younger patients in our cohort) and grade ≥ 3 thrombocytopenia in 3% (versus none in younger patients in our cohort). Of note, relative to our study, Schweigert and coworkers dichotomized their patient cohort using a cut-off of 80 years, while we conducted a dedicated matched-pair comparison focusing on by far younger patients (≤ 65 years), thereby ensuring a broader application of our results in clinical practice. Moreover, in our cohort, all patients with grade 3 toxicities already demonstrated with pre-existing functional impairments at treatment initiation. This is in line with previous studies, focusing on the prediction of adverse events under [^177^Lu]Lu-PSMA RLT, also reporting on an increased rate of adverse events in patients with impaired kidney or bone marrow function [23, 24]. Another potential side effect of therapies including radiation in general are second malignancies [25]. With earlier application of RLT, the risk of second malignancies especially in younger patients with potentially longer remaining life expectancies might gain relevance [7]. Of note, there had been no reports of any second malignancies in younger patients in our study, albeit the small number of patients and limited follow-up period might have limited interpretability for this question.

In a younger patient cohort concerns about health-related quality of life gain even greater importance, as individual expectations for daily functioning may be even higher in this patients group [26]. As part of the VISION trial, *Fizazi* et al. assessed relevant quality of life aspects applying well-established questionnaires such as The Functional Assessment of Cancer Therapy (FACT) scale and the EuroQol 5-dimension 5-level (EQ-5D-5 L) under [^177^Lu]Lu-PSMA RLT compared to the standard of care (SoC) [26, 27]. These instruments enable quantification of functional aspect of everyday life which might be of more relevance in a younger population, including mobility, social functioning and daily activities [26]. This study demonstrated superior quality of life under RLT compared to SoC undermining its well tolerability [26]. These results, together with our findings of strong efficacy and good tolerability in patients ≤ 65 years, further support RLT as a meaningful option for younger subjects.

Some limitations have to be mentioned. Due to the single-center, retrospective trial design, our results need further external validation. We observed a trend toward longer overall survival in younger patients; however, statistical significance was not reached, likely due to the small sample size. Future studies should therefore include a larger patient cohort. Moreover, data about radiographic response was not available in our cohort due to varying follow-up imaging modalities. In addition, patients in our analysis received either [^177^Lu]Lu-PSMA-I&T or [^177^Lu]Lu-PSMA-617. However, comparable results in both dosimetry and outcome analysis between both tracers support the feasibility of a pooled analysis [11, 28]. Notably, a subanalysis stratifying patient into the [^177^Lu]Lu-PSMA-I&T and [^177^Lu]Lu-PSMA-617 cohorts (with five patients in each age group receiving both radiopharmaceuticals) demonstrated results comparable to those of the pooled analysis (Supplemental Tables 1 & 2, Supplemental Fig. 1 & 2). Nonetheless, future prospective trials should ideally use a single standardized agent to enhance comparability.

## Conclusions

[^177^Lu]Lu-PSMA RLT is a safe and effective treatment option in patients ≤ 65 years old. In our matched-pair analysis, younger patients demonstrated comparable efficacy and a slightly beneficial safety profile compared to older patients. As RLT continues to move forward in the therapeutic sequence - supported by recent trials in chemotherapy-naïve and even hormone-sensitive disease - our results strengthen the rationale for a broader and earlier integration of [^177^Lu]Lu-PSMA RLT.

## Supplementary Information

Below is the link to the electronic supplementary material.

Supplementary Material 1 (DOCX 18.3 KB)

Supplementary Material 2 (JPG 233 KB)

Supplementary Material 3 (JPG237 KB)

## Data Availability

The herein presented data is available in case of a reasonable request from the corresponding author.
