# Supplementary material for: [177Lu]Lu-PSMA radioligand therapy in younger prostate cancer patients: A matched-pair analysis between patients ≤ 65 and ≥ 70 years old
Source: Eur J Nucl Med Mol Imaging. 2026 Mar 2;53(8):4873–82. doi: 10.1007/s00259-026-07817-2 (PMC13249676; doi:10.1007/s00259-026-07817-2)
Supplement: Supplementary file 1 — Supplementary Material 1 (DOCX 18.3 KB) [file 259_2026_7817_MOESM1_ESM.docx]

**SUPPLEMENTAL**

**Supplemental Table 1.** Differences of laboratory values during RLT with [^177^Lu]Lu-PSMA-617

| **Laboratory value (%; mean ± SD)** | **≤65 (n=26)** | **≥70 (n=21)** | ***P*** |
| --- | --- | --- | --- |
| PSA change (%) | 34 ± 160 | -10 ± 120 | 0.308 |
| Hb change (%) | -9 ± 11 | -12 ± 11 | 0.299 |
| WBC change (%) | -19 ± 19 | -25 ± 18 | 0.334 |
| Platelets change (%) | -22 ± 14 | -31 ± 22 | 0.098 |
| eGFR change (%) | -4 ± 9 | -5 ± 9 | 0.721 |

RLT, radioligand therapy; Lu, Lutetium; PSMA, prostate-specific membrane antigen; PSA, prostate-specific antigen; Hb, hemoglobin; WBC, white blood cells; eGFR, estimated glomerular filtration rate

**Supplemental Table 2.** Differences of laboratory values during RLT with [^177^Lu]Lu-PSMA-I&T

| **Laboratory value (%; mean ± SD)** | **≤65 (n=20)** | **≥70 (n=25)** | ***P*** |
| --- | --- | --- | --- |
| PSA change (%) | 30 ± 90 | 66 ± 255 | 0.555 |
| Hb change (%) | -6 ± 10 | -14 ± 9 | 0.008 |
| WBC change (%) | -22 ± 23 | -32 ± 13 | 0.099 |
| Platelets change (%) | -21 ± 24 | -34 ± 22 | 0.07 |
| eGFR change (%) | -7 ± 13 | -5 ± 14 | 0.67 |

RLT, radioligand therapy; Lu, Lutetium; PSMA, prostate-specific membrane antigen; PSA, prostate-specific antigen; Hb, hemoglobin; WBC, white blood cells; eGFR, estimated glomerular filtration rate

**SUPPLEMENTAL FIGURE LEGENDS**

**Supplemental Figure 1. Kaplan-Meier analysis of Progression-free survival (PFS) and Overall survival (OS) under [^177^Lu]Lu-PSMA-617 RLT.** RLT demonstrated comparable effectiveness in patients ≤65 years and ≥70 years old, with no statistically significant differences in PFS (**A**) or OS (**B**).

**Supplemental Figure 2. Kaplan-Meier analysis of Progression-free survival (PFS) and Overall survival (OS) under [^177^Lu]Lu-PSMA-I&T RLT.** RLT demonstrated comparable effectiveness in patients ≤65 years and ≥70 years old, with no statistically significant differences in PFS (**A**) or OS (**B**).
