# Supplementary figures and images for: [177Lu]Lu-PSMA radioligand therapy in younger prostate cancer patients: A matched-pair analysis between patients ≤ 65 and ≥ 70 years old
Source: Eur J Nucl Med Mol Imaging. 2026 Mar 2;53(8):4873–82. doi: 10.1007/s00259-026-07817-2 (PMC13249676; doi:10.1007/s00259-026-07817-2)

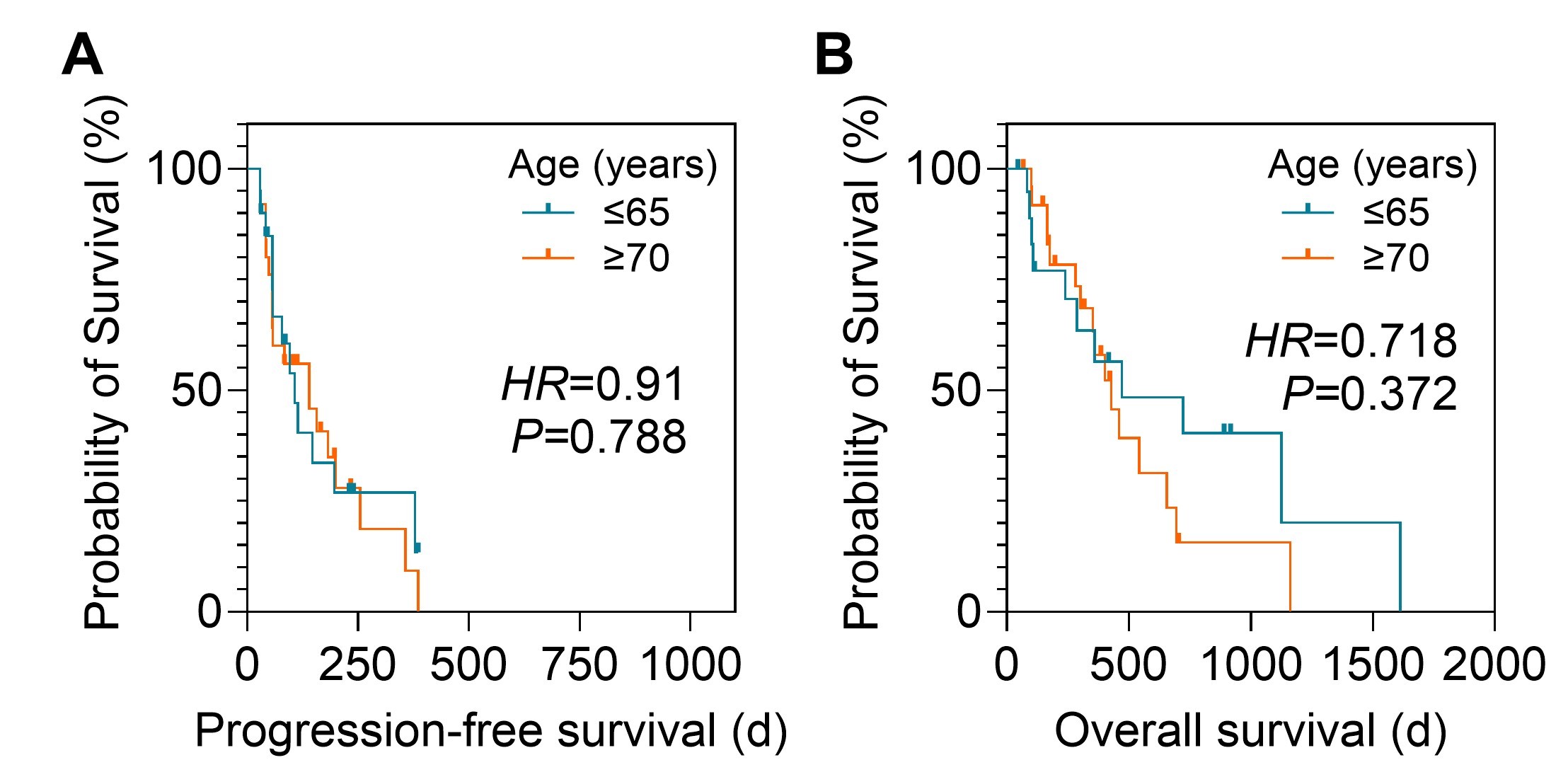

Supplement: Supplementary file 2 — Supplementary Material 2 (JPG 233 KB) [file 259_2026_7817_MOESM2_ESM.jpg]

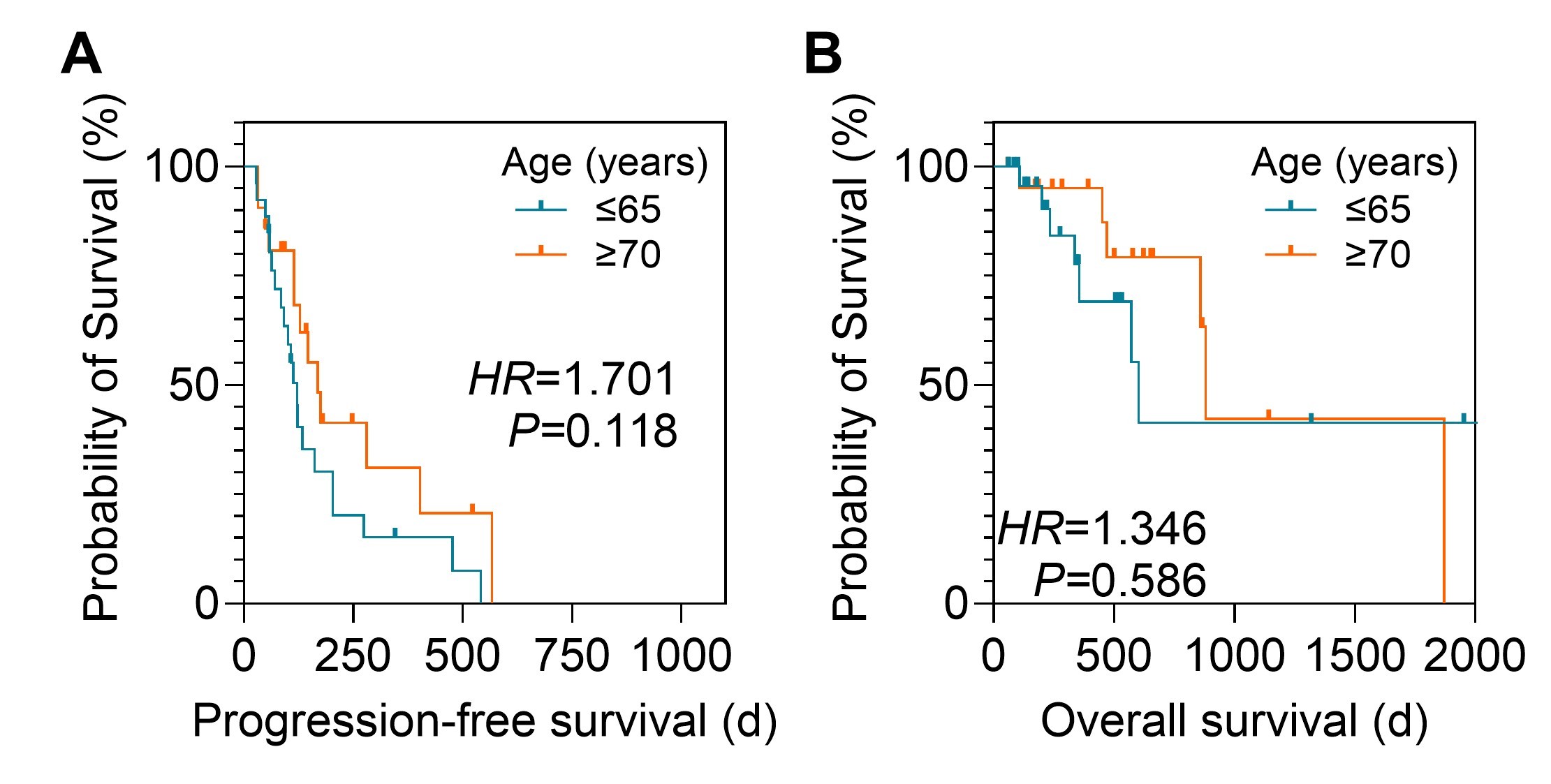

Supplement: Supplementary file 3 — Supplementary Material 3 (JPG237 KB) [file 259_2026_7817_MOESM3_ESM.jpg]
